# Lung organoids: advances in generation and 3D-visualization

**DOI:** 10.1007/s00418-020-01955-w

**Published:** 2021-01-18

**Authors:** Brian Cunniff, Joseph E. Druso, Jos L. van der Velden

**Affiliations:** Department of Pathology and Laboratory Medicine, University of Vermont Cancer Center, Larner College of Medicine, HSRF Bldg., Rm. 214A, Burlington, VT 05405 USA

**Keywords:** Lung biology, Lung organoids, Advanced microscopy, Organoid methodologies

## Abstract

The lung is comprised of more than 40 distinct cell types that support a complex 3-dimensional (3D) architecture that is required for efficient lung function. Loss of this proper architecture can accommodate and promote lung disease, highlighting researchers’ growing need to analyze lung structures in detail. Additionally, in vivo cellular and molecular response to chemical and physical signals, along with the recapitulation of gene-expression patterns, can be lost during the transition from complex 3D tissues to 2D cell culture systems. Therefore, technologies that allow for the investigation of lung function under normal and disease states utilizing the entirety of the lung architecture are required to generate a complete understanding of these processes. Airway cell-derived organoids that can recapitulate lung structure and function ex vivo while being amenable to experimental manipulation, have provided a new and exciting model system to investigate lung biology. In this perspective, we discuss emerging technologies for culturing lung-derived organoids, techniques to visualize organoids using high-resolution microscopy and the resulting information extracted from organoids supporting research focused on lung function and diseases.

**Supplementary Information:**

The online version contains supplementary material available at 10.1007/s00418-020-01955-w.

## Introduction

The lung is one of the most complex organs in the human body, comprised of more than 40 cell types that form a unique architecture to enable its primary function of efficient gas exchange (Franks et al. 2008). The complexity and delicate structure of the lung are primary reasons why acquiring high-quality microscopy images is difficult, particularly three-dimensional (3D) imaging of the lung for studies that require subcellular resolution while maintaining anatomical structure. Over the last decade, cutting-edge microscopic and quantitative histological techniques have been developed for 3D imaging of lung tissue (reviewed in (Schittny 2018)) and, although innovations have been made with regard to lung tissue clearing and preparation, the acquisition of high-quality, clinically relevant data for 3D imaging remains challenging (Gomez-Gaviro et al. 2020; Klouda et al. 2020).

Tremendous progress has been made in culturing 3D organoids from various tissues such as colon (Sato et al. 2011), stomach (Bartfeld et al. 2015), liver and pancreas (Broutier et al. 2016). However, it was not until the beginning of 2019 that long-term lung organoid cultures were reported (Sachs et al. 2019). The development of new 3D culturing techniques has progressed naturally with novel imaging methods, allowing for the characterization of 3D organoid structures to fully comprehend their cellular composition, cell–cell interactions, in situ detection of endogenous proteins, protein modifications and protein interactions. This perspective will briefly discuss the history of lung organoids and summarize the latest developments with respect to their 3D imaging.

## Lung organoids

**Fig. 1:**
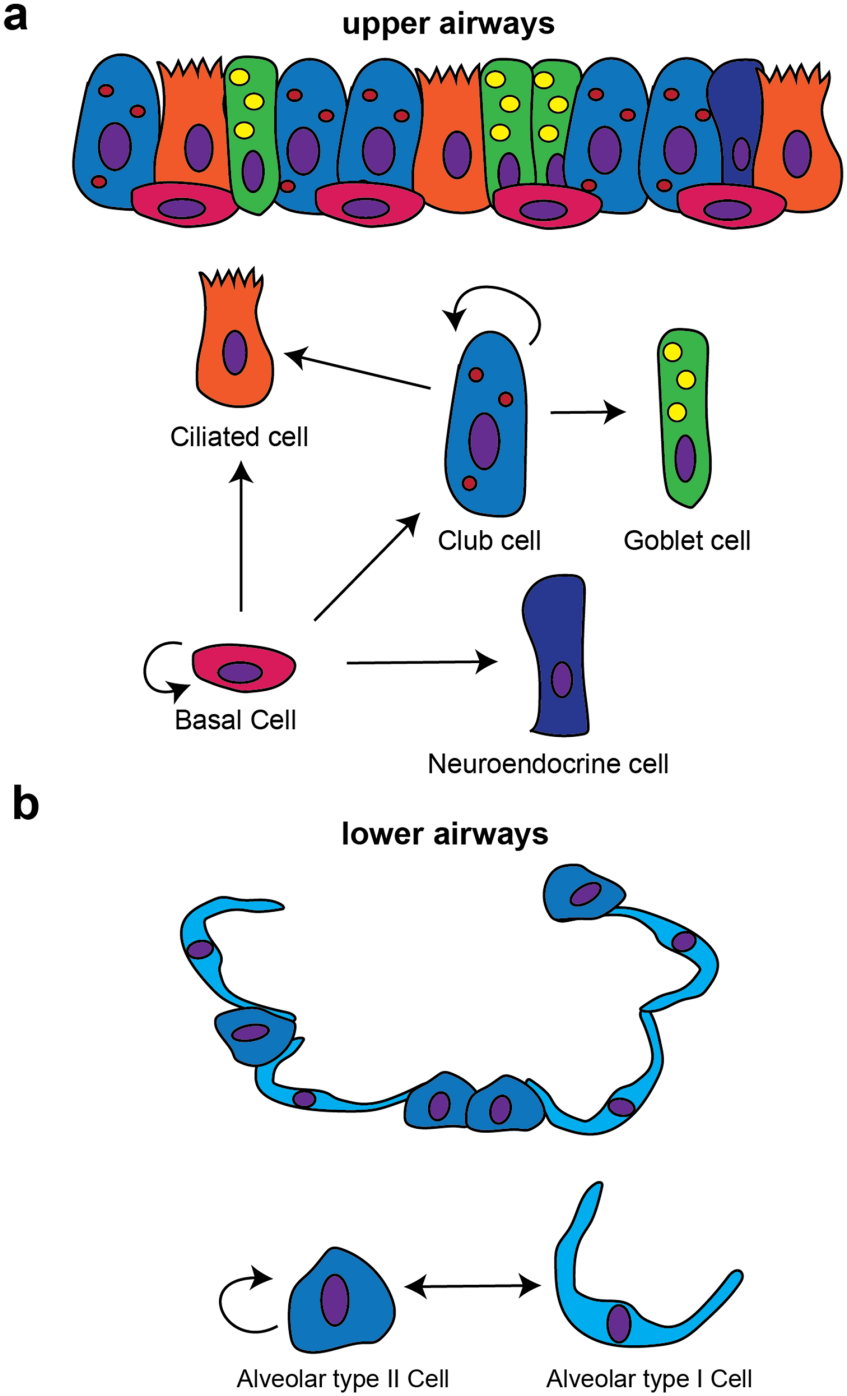
Lung cells with progenitor cell potential. Upper airways (**a**), and lower airways (alveoli) (**b**): adapted from Tata P Development 2017 [17]

Organoids are defined as 3D structures derived from stem/progenitor cells which can recapitulate essential structural and functional aspects of multiple organs, including the lung (Huch and Koo 2015). Since their resurgence in research about a decade ago, organoids are proving to be an essential research tool for applications ranging from fundamental biology to personalized or regenerative medicine. Lung organoids have significant potential in the search for new treatments for almost every lung disease including lung cancer (Kim et al. 2019; Shi et al. 2020), idiopathic pulmonary fibrosis (IPF) (Strikoudis et al. 2019), cystic fibrosis (CF) (Sachs et al. 2019) and asthma (Paolicelli et al. 2019), and recently an in vitro organoid model for human distal lung infectious diseases including COVID-19-associated pneumonia was developed (Salahudeen et al. 2020). Lung organoids have become important tools for researchers to more closely model human diseases such as IPF, as compared to previous models such as the bleomycin mouse model which only shares some gross features of the disease but cannot give insight into the pathophysiology of IPF patients. As many therapeutic drugs that have previously been identified in such preclinical animal models ultimately fail in human clinical trials, there is well-founded hope that organoid model systems will result in novel therapeutic discoveries with lower drug attrition rates. Numerous approaches have been investigated and developed to culture lung organoids, reviewed in (Barkauskas et al. 2017). As organoids are derived from cells with progenitor potential, lung organoids can be derived from epithelial stem/progenitor cell populations found within the adult lung, which qualifies three types of cells. In the upper and middle airways, basal cells and secretory club cells (previously named Clara cells) (Fig. 1a), and in the lower airways alveolar type II cells (AEC2) cells (Fig. 1b).

**Fig. 2:**
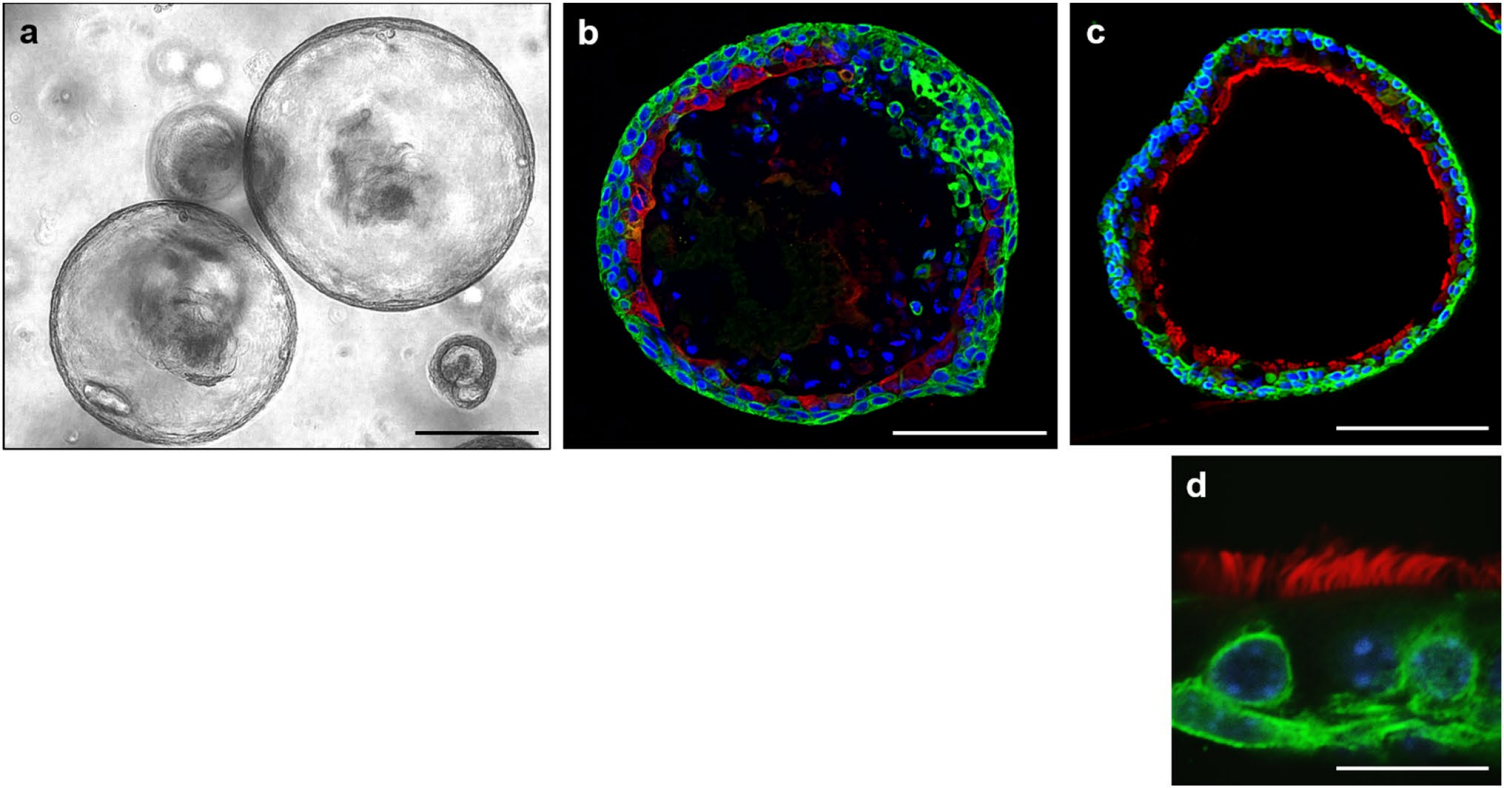
Tracheospheres derived and mouse tracheal basal cells and grown in 3D conditions are comprised of multiple airway cell types. **a** Brightfield image of live tracheospheres in 3D culture. **b** Immunofluorescence staining of 5 µm paraffin embedded sections from tracheospheres stained for basal marker cytokeratin 5 (green), lumenal cell marker cytokeratin 8 (red) and nuclei (blue). **c** Immunofluorescence staining of 5 µm paraffin embedded sections from tracheospheres stained for basal marker cytokeratin 5 (green), ciliated cell marker acetylated tubulin (red) and nuclei (blue). **d** Magnified image of immunofluorescence staining of 5 µm paraffin embedded sections from tracheospheres stained for basal marker cytokeratin 5 (green), ciliated cell marker acetylated tubulin (red) and nuclei (blue) as seen in (**c**). Scale bars are 100 µm in (**a**–**c**), 10 µm in (**d**)

Basal cells being positive for markers such as tumor protein p63 (TRP63) and cytokeratin 5 (KRT5) are stem/progenitor cells that maintain airway homeostasis and the first organoids derived from mouse tracheal basal cells were referred to as tracheospheres (Rock et al. 2009). In the human lung, TRP63 + and KRT5 + basal cells exist throughout the airways, hence basal organoids derived from the trachea are named tracheospheres and basal organoids derived from the large airways are called bronchospheres. These organoid model systems have provided researchers the opportunity to study the growth and differentiation of airway basal cells into secretory or ciliated cell lineages in a 3D tissue-like environment (Fig. 2) and offer a powerful research platform from which to study human airway disease. However, the mixed population of airway secretory cells, together with their extraordinary ability to dedifferentiate or generate other airway lineages ranging from basal to AEC (both 1 and 2) cells (Tata and Rajagopal 2017), has made it difficult to classify airway secretory cell-derived organoids. Therefore, additional cell-type markers and improved culture conditions are needed to allow more detailed analyses of organoids derived from airway secretory cells.

The alveolar epithelium of the lung is composed of two distinct epithelial cell types. Alveolar epithelial type II cells (AEC2) are characterized by the production of pulmonary surfactant proteins, while alveolar epithelial type I cells (AEC1) cover most of the surface area of the alveoli and perform the function of gas exchange. Interestingly, AEC2 cells can form alveolospheres and differentiate into organoid structures that contain both AEC2s and AEC1s (Barkauskas et al. 2013).

A fourth option is the generation of lung organoids derived from human pluripotent stem cells (hPSCs), which includes embryonic stem cells and induced pluripotent stem cells (iPSCs). The development of human pluripotent stem cell (hPSC)-derived organoids (McCauley and Wells 2017) has enabled lung organoids derived from hPSC to be studied (Huang et al. 2015; Chen et al. 2017), with the added advantage that hPSCs can be manipulated, for example, by CRISPR/Cas9 gene editing (Strikoudis et al. 2019). This technology has enabled researchers to examine human genes associated with disease and development in lung organoids.

Further details summarizing the different methods for generating organoids from human and mouse lungs can be found in an overview by (Barkauskas et al. 2017). Until recently, most of these methods did not allow for prolonged expansion of lung organoids from adult human individuals, but the lung organoid field is evolving rapidly. Sachs et al. reported long-term culture conditions for human lung tissue-derived lung organoids, these organoids were passaged every other week for over 1 year, while retaining similar frequencies of basal, club, multi-ciliated, and secretory cell (Sachs et al. 2019). Also, lung organoids generated from hPSCs can be cultured for an extended time periods of up to 170 days (Chen et al. 2017). Another major step was made when Salahudeen et al. described a new, robust method for long-term human distal lung airway and alveolar organoid growth (Salahudeen et al. 2020).

## Lung cancer organoids

In vivo lung cancer experiments have proved challenging and 2D lung cancer cultures have substantial limitations in replicating in vivo tumor characteristics. However, recent developments in 3D organoid culturing techniques has created new possibilities for the development of physiologically relevant human cancer models (Drost and Clevers 2018). Cancer organoids have valuable advantages over other cancer model systems, such as patient-derived xenograft models (PDXs), in that they require less time to establish while stably maintaining morphological and genetic tumor features even after long-term expansion (Sato et al. 2011, Sachs et al. 2018). Recent exciting studies showed that lung cancer organoids recapitulate the tissue architecture of primary lung tumors and maintain the genomic alterations of the original tumors during long-term expansion in vitro (Kim et al. 2019; Shi et al. 2020). However, caution must be taken since normal lung organoids can overgrow lung tumor organoids (Dijkstra et al. 2020). Although not technically a lung-cancer, malignant mesothelioma patient-derived tumor organoids have been used as a screening platform, accurately predicting patient response to tailored therapeutics (Mazzocchi et al. 2018).

Therefore, tumor-derived organoids are an excellent alternative in vitro model that retains the characteristics of the original tumor and can potentially serve as a platform for biobanking and selecting personalized therapeutic approaches (Vlachogiannis et al. 2018). Techniques to generate, maintain and biobank lung organoids have increased tremendously over the past couple of years and it is expected that organoid culture will become an indispensable tool for both basic and applied lung research.

## 3D imaging of organoids

The advantages of 3D culture systems over 2D systems have become evident (Fong et al. 2016, Avnet et al. 2019, D'Agosto et al. 2019, Yang et al. 2019), especially the value of organoids in cancer research. As discussed, over the last couple of years organoids as a model to study various lung diseases has experienced incredible development. However, most organoid analysis is limited to conventual microscopy and biochemical analysis, such as end-point metabolic ATP assays that totals the responses of all the cells in an organoid. With the rapid expansion of the number of research groups using organoid applications and their increasing complexity, rapid, cost-effective and user-friendly 3D imaging approaches to fully capture the potential and complexity of organoid cultures is required.

**Fig. 3:**
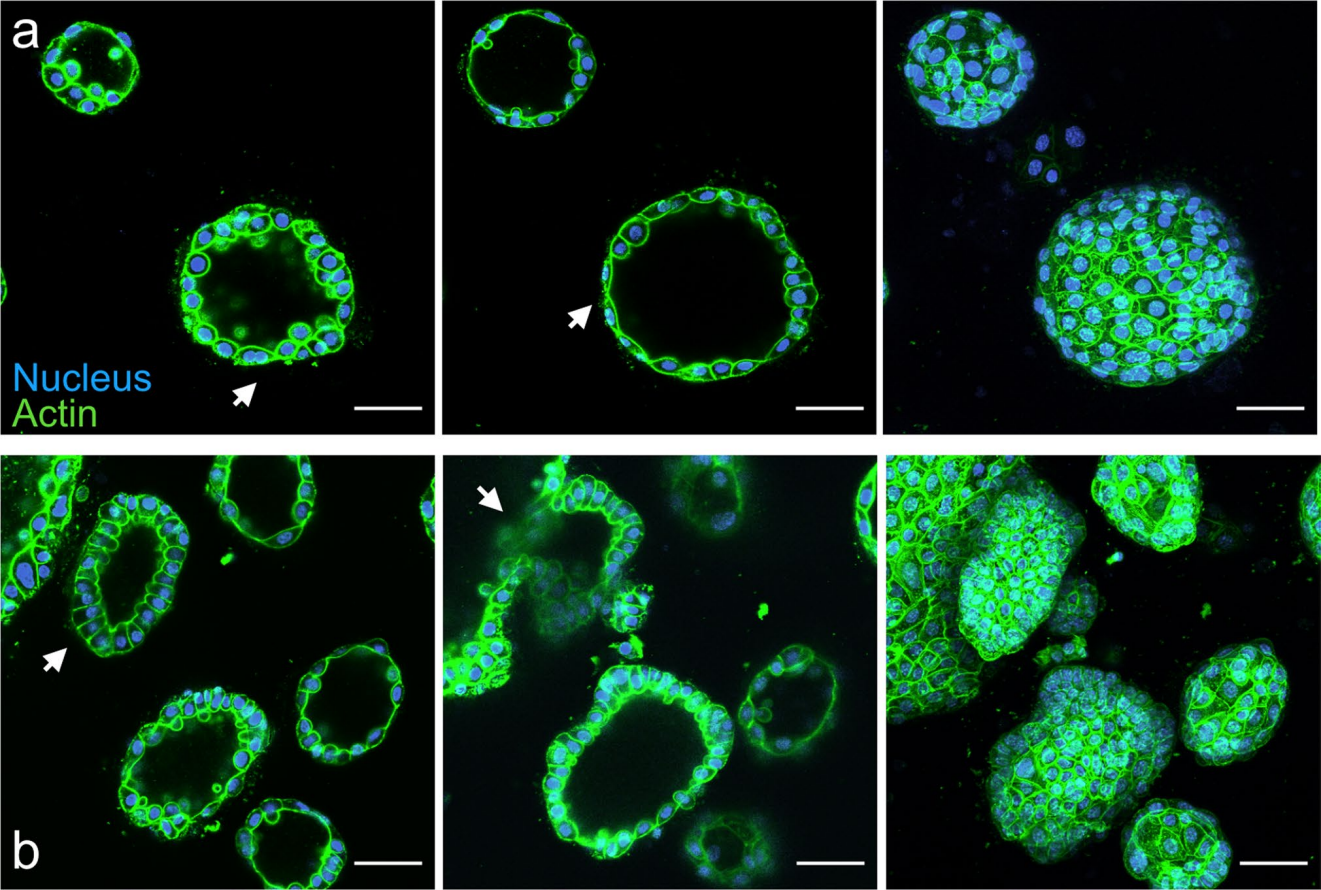
Lung organoids derived and cultured from tumor bearing mouse lung lobes. Organoids were cultured for 7 days before fixation and staining with Alexa-488 phalloidin and DAPI. Optical Sects. (2 µm thickness) were collected at the Microscopy Imaging Center at the University of Vermont on a Nikon A1-R laser scanning confocal microscope (RRID# SCR_018821) supported by NIH award number 1S10OD025030-01 from the National Center for Research Resources **a** Spherical organoid with mostly non-polarized cells surrounding a cleared lumen. Left and middle are optical sections separated by 15 µm, right is a max intensity projection of 95 optical sections. **b** Spherical and non-spherical lung organoids with the majority of cells showing a polarized phenotype. Left and middle are optical sections separated by 15 µm, right is a max intensity projection of 95 optical sections. Scale bar = 100 µm

Noninvasive microscopy methods such as confocal, multiphoton laser scanning microscopy and light-sheet fluorescence microscopy (LSFM) make it possible to visualize cellular details as well as overall tissue architecture within a single biological sample. Using organoid cultures derived from tumor bearing mouse lungs, and laser scanning confocal microscopy, a detailed yet heterogenous architecture can be observed in 3D (Fig. 3 and Supplemental Video 1). Both polarized and non-polarized cells can be visualized in spherical structures and in more complex protrusions from larger 3D structures (Fig. 3 and Supplemental Video 1). It appears as polarized and non-polarized cell types are restricted to their individual organoid, an observation requiring more detailed evaluation. Post-processing analysis software provides methodology to evaluate organoid architecture and cell composition and protein expression patterns across sample types following imaging with standard 3D confocal approaches. Long-term, live cell-imaging of organoid growth can be achieved using specialized environmental chambers and non-invasive imaging approaches, but as organoids require increased attention while growing compared to 2D cell culture, challenges still exist for capturing ex vivo organoid growth (Rios and Clevers 2018).

## Clearing of organoids

Recently, a detailed protocol for high-resolution 3D imaging of fixed and cleared organoids was published (Dekkers et al. 2019) which was followed up by a refined protocol (van Ineveld et al. 2020) utilizing a new nontoxic clearing agent FUnGI (Rios et al. 2019). Clearing techniques have been developed to minimize aberrations generated from the change in refractive index between the sample and the microscope objective, referred to as light scattering (Richardson and Lichtman 2015, 2017). Earlier clearing approaches such as DISCO (Erturk et al. 2012), CUBIC (Murakami et al. 2018), and CLARITY (Tomer et al. 2014) were mostly developed for large, intact tissues and require long incubation times, preventing rapid screening under different experimental conditions. Recently developed clearing techniques are more user-friendly and provide a simple, efficient method to better visualize and examine organoids (Gomez-Gaviro et al. 2020). Another simple and robust clearing method for complex 3D structures named 2Eci (2nd generation ethyl cinnamate-based clearing) was developed to preserve fluorescent proteins while being compatible with antibody staining and in situ hybridization (Masselink et al. 2019). This enables multiplexed microscope approaches to evaluate organoid architecture. Most clearing techniques are designed to improve penetration of fixed organoids but there is also a need for clearing methods that maintain cell viability, for example, to enable drug screening over time. An extensive overview of clearing techniques for imaging of 3D structures was provided by (Costa et al. 2019). One of the compounds recently identified that enables live imaging was Iodixanol, which improved image quality in live human cerebral organoid experiments (Boothe et al. 2017).

## High-throughput live imaging of organoids

The fact that organoids can be cultured and expanded in vitro reveals new opportunities for high-throughput imaging analysis for both research and personalized medicine approaches. An example of rapid high-throughput personalized medicine was a fluorescence-based swelling assay in organoids from cystic fibrosis patients which facilitated diagnosis and drug development in cystic fibrosis (Dekkers et al. 2013). There are some limitations to this, in that live fluorescent imaging of 3D organoid cultures with conventional laser scanning microscopy is difficult because of a low acquisition speed, decreased resolution and light scattering within the tissue (Ntziachristos 2010). Recent advances in light-sheet fluorescence microscopy (LSFM) provide imaging capabilities with increased acquisition speed, optical sectioning and a desirable signal-to-noise ratio (Power and Huisken 2017). An example of a high-throughput screening utilizing light-sheet microscopy was developed by Eismann et al. where they developed a protocol for automated evaluation of mitotic phenotypes in 3D cultures (Eismann et al. 2020).

## Conclusions

The lung is a critically important organ that allows for the proper exchange of high volumes of gas between our bodies and the environment. To accomplish this feat, the lung has evolved as an efficient, durable and intricate organ that requires the concerted actions of many cell types to maintain normal function. The high level of complexity found in lung tissue composition, architecture and function demands advanced techniques for accurate modeling and observation. The 3D organoid model system provides a unique advantage to disease investigators in that it provides the opportunity to closely analyze physiologically relevant lung development or disease progression in vitro. To do this, however, many investigators have found an increasing need for high-quality, high-throughput and affordable imaging. Traditional imaging techniques that employ paraffin embedding and slicing of tissue into thin layers do not allow for the subsequent analysis of larger scale organ structures. In response to this limitation, 3D rendering of tissues and organoids has become a central focal point for microscopic imaging. However, while there is a rapidly growing demand for imaging 3D organoids, the thickness of these structures can cause light scattering due to mismatched refractive indices within the heterogenous sample of cells. Because of this, optical clearing methods have been developed that employ sugars and/or glycerol to reduce scattering within the sample.

Collectively, these advancements in generation of lung organoids and imaging technologies have enabled organoid model systems to propel lung development and disease research forward and offer exciting, novel therapeutic insights.

## Supplementary Information

Below is the link to the electronic supplementary material.

Supplementary file1 (AVI 1234 KB) Supplementary Video 1: Stepwise transition through 2 µm thick optical sections of tumor bearing mouse derived lung organoids. Note spherical organoids that are an extended protrusion from larger airways.
